# Consecución de objetivos farmacocinéticos/farmacodinámicos en pacientes críticos con monitorización terapéutica de linezolid

**DOI:** 10.23938/ASSN.1151

**Published:** 2026-03-31

**Authors:** Irati Irigoyen Rodriguez, Leire Leache, Julen Fernández-González, Amaia Egües, Julián Librero López

**Affiliations:** 1 Servicio Navarro de Salud Hospital Universitario de Navarra Servicio de Farmacia Pamplona España; 2 Servicio Navarro de Salud Subdirección de Farmacia y Prestaciones Servicio de Gestión de la Prestación Farmacéutica Pamplona España; 3 Servicio Navarro de Salud Subdirección de Farmacia y Prestaciones Servicio de Asesoría e Información del Medicamento Pamplona España; 4 Navarrabiomed. Unidad de Metodología. Pamplona. España. Navarrabiomed Unidad de Metodología Pamplona España; 5 Red de Investigación en Cronicidad, Atención Primaria y Promoción de la Salud (RICAPPS), Instituto de Salud Carlos III. España. Red de Investigación en Cronicidad, Atención Primaria y Promoción de la Salud (RICAPPS) Instituto de Salud Carlos III España

**Keywords:** Monitoreo de Drogas Terapéuticas, Farmacocinética, Linezolid, Unidad de Cuidados Intensivos, Therapeutic Drug Monitoring, Pharmacokinetics, Pharmacodynamics, Linezolid, Intensive Care Unit

## Abstract

**Introducción:**

La pauta habitual de linezolid se asocia con exposición subóptima en pacientes críticos. En este estudio se analiza la asociación entre las distintas modalidades de tratamiento (dosis, tiempo de infusión) y la probabilidad de alcanzar los objetivos farmacocinéticos/farmacodinámicos, evaluando su impacto microbiológico y clínico.

**Pacientes y métodos:**

Estudio retrospectivo en pacientes ingresados en unidades de cuidados intensivos con monitorización terapéutica de linezolid entre mayo de 2021 y diciembre de 2023. Se analizó el cumplimiento de los objetivos farmacocinéticos/farmacodinámicos (Cmin: 2-7 mg/L, AUC: 0-24/CMI: 80-120 mg*h/L, y T: >CMI ≥85%) en función de la pauta inicial (1.200 o 1.800 mg/día) y tiempo de infusión (1 o 3 h). Se analizaron subgrupos de riesgo: obesidad, aclaramiento de creatinina aumentado, técnicas de reemplazo renal y <65años.

**Resultados:**

Se incluyeron 70 pacientes. En la primera monitorización, el 18,6% alcanzó los tres objetivos farmacocinéticos/farmacodinámicos y el 40% tuvo la Cmin en rango. No hubo diferencias estadísticamente significativas en la consecución de objetivos según la pauta o el tiempo de infusión (tampoco en subgrupos de riesgo). Sin embargo, el cumplimiento de Cmin fue superior con la dosis de 1.800 mg/día (54,5% vs. 37,3%). Tampoco se detectaron diferencias significativas respecto a resultados microbiológicos y clínicos según Cmin estuviese en rango o no.

**Conclusiones:**

Menos del 20% de pacientes alcanzó todos los objetivos farmacocinéticos/farmacodinámicos con la pauta inicial. Estos resultados sugieren la necesidad de individualizar y monitorizar las pautas para optimizar el tratamiento en esta población, especialmente en perfiles de riesgo.

## Abstract (en)

**Background:**

Standard dosing of linezolid regimen frequently results in suboptimal exposure in critically ill patients. This study aimed to analyze the association between dosing regimens (total daily dose and infusion duration) and the probability of achieving pharmacokinetic/pharmacodynamic targets, as well as their microbiological and clinical impact.

**Methods:**

A retrospective observational study was conducted in cri-tically ill patients admitted to intensive care units who underwent therapeutic drug monitoring of linezolid between May 2021 and December 2023. Achievement of pharmacokinetic/pharmacodynamic targets (Cmin: 2-7 mg/L, AUC: 0-24/MIC: 80-120 mg*h/L, and T: >MIC ≥85%) was evaluated according to the initial regimens (1,200 or 1,800 mg/day) and infusion duration (1 or 3 hours). Risk subgroups included obesity, augmented creatinine clearance, renal replacement therapy, and age <65 years.

**Results:**

Seventy patients were included. At the first monitoring, only 18.6% achieved all three pharmacokinetic/pharmacodynamic targets and 40% achieved the Cmin target. No statistically significant differences were observed in target attainment according to dose or infusion duration, including within risk subgroups. However, the 1,800 mg/day regime showed a numerically higher proportion of patients achieving the Cmin target (54.5% vs. 37.3%). No significant differen-ces in microbiological or clinical outcomes were observed between patients with Cmin within the target range and those outside it.

**Conclusions:**

Fewer than 20% of critically ill patients achieve all pharmacokinetic/pharmacodynamic targets with the initial regimen. These findings support the need for individualized dosing and therapeutic drug monitoring to optimize linezolid treatment in this population, particularly in high-risk patients.

## INTRODUCCIÓN

Los pacientes críticos presentan alteraciones farmacocinéticas (metabolismo y/o excreción, volumen de distribución, unión a proteínas plasmáticas, eliminación, etc.) que, unidas a otros factores, hacen que el empleo de las dosificaciones convencionales pueda conllevar fracaso clínico^1^. Por ello, la monitorización terapéutica de medicamentos antimicrobianos (TDM, *therapeutic drug monitoring*) aporta un gran valor al permitir individualizar las pautas de dosificación para alcanzar los objetivos farmacocinéticos/farmacodinámicos (PK/PD), optimizando así la eficacia clínica y minimizando los riesgos de toxicidad o resistencias.

Linezolid es una oxazolidinona ampliamente utilizada en pacientes críticos^2^^,^^3^; la pauta posológica indicada en ficha técnica es de 600 mg/12h por vía oral o intravenosa^4^. Es un fármaco lipofílico con baja unión a proteínas cuya eliminación ocurre vía excreción renal (35%) o por vías enzimáticas^5^. Sus concentraciones plasmáticas muestran una elevada variabilidad intra e interindividual, lo que puede propiciar desde una posible infradosificación y, por tanto, falta de efectividad y aparición de resistencias; hasta una sobredosificación y toxicidad^1^^,^^6^.

La máxima eficacia se ha demostrado cuando el tiempo durante el que las concentraciones de linezolid permanecen por encima de la concentración mínima inhibitoria (CMI) es, como mínimo, un 85% del tiempo intervalo posológico (T>CMI ≥85%), y con una ratio del área bajo la curva respecto a la CMI (AUC 0-24/CMI) de 80-120 mg*h/L^1^. Pero también se ha descrito trombocitopenia inducida por linezolid a una concentración mínima (Cmin) >7-10 mg/L y AUC 0-24 >300-350 mg*h/L^1^. Por ello, se recomienda la consecución de los parámetros PK/PD a fin de garantizar una exposición óptima mientras se reduce el riesgo de toxicidad hematológica^1^.

En pacientes críticos se observa un mayor aclaramiento plasmático y la posología estándar resulta subóptima en muchos casos^8^^-^^14^. Por ello, los pacientes críticos suelen beneficiarse de dosis de linezolid superiores a las estándar y/o de infusiones prolongadas, aunque estas estrategias deberían estar guiadas por la TDM^1^^,^^5^^,^^15^.

Según la evidencia disponible^1^, en ciertos perfiles de pacientes ingresados en la Unidad de Cuidados Intensivos (UCI) (pacientes con obesidad, con aclaramiento de creatinina aumentado, con aislamientos microbiológicos con CMI ≥2 mg/L, con síndrome de distrés respiratorio agudo, etc.) el tratamiento con linezolid se inicia con dosis intensificadas o con prolongación de la duración de la infusión, al menos hasta la realización de la TDM. De acuerdo- a la bibliografía disponible, el tiempo de infusión de linezolid intravenoso se establece por defecto en 3 horas en todos los pacientes que reciben la pauta estándar de 600 mg/12h^6^.

El objetivo del estudio consiste en analizar la asociación entre las distintas modalidades de tratamiento de linezolid (en cuanto a dosis y tiempo de infusión) y la probabilidad de que se alcancen los objetivos terapéuticos en los parámetros PK-PD en pacientes críticos. También se evaluó el impacto de alcanzar el objetivo terapéutico PK-PD en los resultados microbiológicos y clínicos.

## MATERIAL Y MÉTODOS

### Diseño del estudio y población

Se llevó a cabo un estudio observacional transversal en personas ingresadas en las unidades de cuidados intensivos (UCI) del Hospital Universitario de Navarra (HUN) en Pamplona (España), a las que se les realizó monitorización terapéutica de linezolid entre mayo de 2021 y diciembre de 2023. No hubo ningún criterio de exclusión.

El estudio contó con la autorización del Comité de ética de la Investigación con medicamentos de Navarra (EO-204/16).

### Monitorización terapéutica de linezolid y objetivos PK/PD

La primera TDM se realizó tras la quinta dosis de linezolid recibida, para asegurar haber alcanzado el estado estacionario. En caso de modificarse la pauta posológica tras la TDM, la siguiente monitorización se realizará tras recibir un mínimo de cinco dosis con la nueva pauta. En caso de cumplir los objetivos PK/PD y mantener la misma pauta, se realiza una nueva determinación PK/PD si la situación clínica del paciente se modifica considerablemente o si el tratamiento se prolonga durante más de siete días desde la monitorización.

En los informes emitidos por parte del Servicio de Microbiología del HUN, se informa acerca de la sensibilidad o resistencia de los microorganismos a linezolid tomando como referencia los puntos de corte que establece la *European Committee on Antimicrobial Susceptibility Testing* (EUCAST), siendo el punto de corte más común para la mayoría de bacterias Gram-positivas una CMI de 4 mg/L. Sin embargo, teniendo en cuenta la epidemiología local y las discrepancias entre los puntos de corte de EUCAST y el *Clinical & Laboratory Standards Institute* (CLSI), para calcular los objetivos PK/PD (AUC 0-24/CMI y T>CMI) se consideró un valor de CMI de 2 mg/L.

### Información y variables del estudio

Toda la información para el estudio se extrajo de la historia clínica informatizada (HCI), y se analizó de forma anonimizada. Se obtuvieron las siguientes variables:

- demográficas: edad (años), sexo;
- clínicas: índice de masa corporal (IMC) en kg/m^2^, bilirrubina total y transaminasas, aclaramiento de creatinina (mL/min) en pacientes sin terapia de reemplazo renal, técnicas de reemplazo renal; inmunodepresión, tratamiento concomitante con inductores y/o inhibidores potentes del citocromo CYP3A4; aislamientos microbiológicos (bacteria) y localización, shock séptico, puntuación en la escala *Sequential Organ Failure Assessment* (SOFA);
- tratamiento con linezolid: empírico o dirigido, posología, vía de administración, tiempo de infusión (1 o 3 h).

Las variables de resultado fueron:

- cumplimiento de los objetivos ya mencionados en los parámetros PK-PD (Cmin entre 2-7 mg/L, T>CMI ≥85%, AUC 0-24/CMI entre 80-120 mg*h/L);
- resultados clínicos: curación clínica, duración de estancia en UCI, duración de la estancia hospitalaria, mortalidad a 30 días;
- resultados microbiológicos: negativización del cultivo, tiempo hasta la negativización del cultivo, recaída o nueva infección a 30 días.

### Análisis estadístico

Se describieron las características basales de los pacientes, las categóricas mediante frecuencias y porcentajes y las cuantitativas como media y desviación estándar (DE) o como mediana y rango intercuartílico (RIC) (P_75_-P_25_).

Se analizaron los resultados en relación al cumplimiento de los objetivos en los parámetros PK-PD, microbiológicos y clínicos. A su vez, se analizaron los principales resultados en función de las características basales (demográficas y clínicas), para identificar las características basales que pudiesen impactar de manera significativa en los resultados. También se analizó la probabilidad de tener la Cmin de linezolid en rango terapeútico en la primera monitorización en función de la dosis diaria inicial de linezolid en cuatro subgrupos específicos de pacientes (con IMC >30 kg/m^2^, con aclaramiento de creatinina >120 mL/min, con terapia de reemplazo renal, y en menores de 65 años).

Se analizaron las siguientes asociaciones: haber alcanzado los objetivos en los parámetros PK/PD en la primera monitorización (variable dependiente) en función de la pauta posológica inicial de linezolid y del tiempo de infusión del mismo (variables independientes); el requerimiento del cambio de modalidad de tratamiento si la Cmin no se encontraba en rango tras cualquier monitorización (variable dependiente) en función de la pauta posológica de linezolid (variable independiente), los resultados microbiológicos (variables dependientes) en función de si la Cmin estaba o no en rango en algún momento durante el tratamiento (variable independiente), y los resultados clínicos (variables dependientes) en función de si la Cmin estaba o no en rango en algún momento durante el tratamiento (variable independiente).

Se realizó un análisis bivariante para evaluar la asociación entre la pertenencia al grupo definido por cada pregunta del estudio y el resto de las variables de interés. Para las variables nominales, la asociación se analizó mediante la prueba de Chi-cuadrado de Pearson, utilizando el test exacto de Fisher en aquellos casos donde las frecuencias esperadas fueron inferiores a 5. En el caso de las variables cuantitativas (continuas y discretas), se evaluó inicialmente su normalidad mediante la prueba de Shapiro-Wilk. Para variables con distribución normal, se empleó la prueba t de Student; por el contrario, para variables con distribución no paramétrica o de naturaleza discreta con rangos limitados, la mayoría, se utilizó la prueba U de Mann-Whitney (o Wilcoxon para muestras independientes).

La asociación se evaluó mediante análisis bivariante y, en caso de obtener resultados estadísticamente significativos, se realizaron análisis de regresión logística multivariante ajustados por las características basales (demográficas y/o clínicas) de pacientes. Los resultados de variables categóricas se informaron como *odds ratio* cruda (OR) -y ajustada (ORa) cuando correspondía- con su correspondiente intervalo de confianza del 95% (IC95%) y, en el caso de variables cuantitativas, como diferencia de medias (DM) junto con su IC95%.

Se consideró como diferencia estadísticamente significativa una p<0,05 para un contraste bilateral. Los análisis estadísticos se realizaron mediante el software R®.

## RESULTADOS

Se incluyeron 70 pacientes ingresados en las UCI, tratados con linezolid y sometidos a TDM durante el periodo de estudio. Predominaron los hombres (64,3%), la mediana de edad fue 60,5 (rango: 15-86) años y la mediana de IMC estuvo en el rango de obesidad. Un 25,7% de los pacientes recibió tratamiento con inhibidores potentes del CYP3A4 y un 27,1% tenía la bilirrubina total elevada. Los pacientes mostraron mayormente infección de localización intraabadominal (50,0%) o respiratoria (24,3%) (Tabla 1).

**Tabla 1:** Características basales de la muestra de pacientes del estudio

*Variables demográficas, analíticas y clínicas*	
Edad (años), mediana (RIC)	60,5 (17,8)
Sexo (mujer), n (%)	25 (35,7)
Índice de masa corporal (kg/m^2^), mediana (RIC)	31,5 (8,1)
Terapia de reemplazo renal, n (%)	11 (15,7)
Aclaramiento de creatinina (mL/min)^a^, mediana (RIC)	97,1 (67,5)
Bilirrubina total (mg/dL), mediana (RIC)	0,6 (0,8)
Bilirrubina total >1,2 mg/dL, n (%)	19 (27,1)
Transaminasas >3 veces LSN, n (%)	11 (15,7)
*Pacientes con inhibidores o inductores potentes del CYP3A4, n (%)*	
*Total*	*22 (31,4)*
Inhibidores potentes del CYP3A4^b^	18 (25,7)
Inductores potentes del CYP3A4^c^	4 (5,7)
Inmunodepresión	10 (14,3)
*Diagnóstico infeccioso, n (%)*	
Intraabdominal	35 (50,0)
Tracto respiratorio	17 (24,3)
Piel y partes blandas	7 (10,0)
Tracto urinario	1 (1,4)
SNC	2 (2,9)
Bacteriemia	2 (2,9)
Artritis séptica	1 (1,4)
Varios	2 (2,9)
Desconocido	3 (4,3)
Pacientes con shock séptico, n (%)	32 (45,7)
SOFA^d^, mediana (RIC)	9 (3,25)

RIC: rango intercuartílico; LSN: límite superior de la normalidad; a: solo pacientes sin terapia de reemplazo renal (n=59); b: amiodarona, fluconazol, ritonavir, voriconazol; c: rifampicina; d: solo pacientes con shock séptico que disponían de puntuación en la escala *Sequential Organ Failure Assessment* (SOFA) (n=24).

La mayoría de tratamientos con linezolid se iniciaron de manera empírica (82,9%), todos por vía intravenosa, generalmente con dosis diaria inicial de 1.200 mg (84,3%) y tiempo de infusión en la pauta inicial de 3 horas (68,6%). La mediana de la duración del tratamiento fue de 13 días (RIC=11,8). Casi la mitad de pacientes (n=31; 44,3%) presentaron aislamientos susceptibles de tratarse con linezolid al inicio o durante el tratamiento con el mismo, nueve de los cuales (29%) tuvieron más de un aislamiento. Los aislamientos más frecuentes correspondían a los géneros *Enterococcus* y *Staphylococcus*, seguidos por *Streptococcus*. Todas las cepas aisladas fueron sensibles a linezolid con una CMI ≤2 mg/mL (Tabla 2).

**Tabla 2:** Aislamiento de microorganismos susceptibles al tratamiento inicial con linezolid

Microorganismo aislado	Pacientes
*Enterococcus sp.*	17
*E. faecium*	11
*E. faecalis*	3
*E. casseliflavus*	1
*E. avium*	2
*Staphylococcus sp*	16
*S. aureus* sensible a meticilina	5
*S. aureus* resistente a meticilina	4
*S. epidermidis*	4
*S. hominis*	1
*S. capitis*	1
*S. haemolyticus*	1
*Streptococcus sp.*	8
*S. anginosus*	3
*S. pyogenes*	2
*S. mitis*	1
*S. salivarus*	1
*S. parasanguilis*	1
*Enterobacter asburicae*	1
*Cutibacterium acnes*	1
*Fusobacterium necrophorum*	1

La información correspondiente a todas las TDM de linezolid realizadas en todos los pacientes y a los resultados obtenidos en los distintos objetivos se muestra en la Tabla 3. En al menos una de las monitorizaciones de linezolid se observaron desviaciones de los objetivos PK/PD en un número relevante de pacientes: 37,1% respecto a la Cmin, la gran mayoría por presentar valores inferiores a 2 mg/L; 57,1% respecto a la relación AUC 0-24/CMI, generalmente por valores inferiores a 80 mg·h/L, y casi un tercio de pacientes tuvieron un T>CMI <85% (Tabla 3). Debe considerarse que un mismo paciente pudo encontrarse dentro del rango terapéutico en una monitorización y fuera de rango en otra. La Tabla 3 también muestra los resultados de la TDM en relación con la pauta posológica y el tiempo de infusión de linezolid.

Un 40% de los pacientes tuvieron la Cmin de linezolid en rango terapéutico en la primera monitorización con la pauta inicial frente al 18,6% que tuvieron los tres parámetros PK/PD (Cmin, el AUC 0-24/CMI y el T>CMI). No se hallaron diferencias significativas en los tres parámetros PK-PD en relación a la dosis diaria inicial de linezolid ni en función del tiempo de infusión. Sin embargo, seis de los 11 pacientes (54,5%) que recibieron una pauta inicial de linezolid de 1.800 mg/día presentaron la Cmin en rango en la primera monitorización, frente a los 22 de los 59 pacientes (37,3%) que recibieron inicialmente 1.200 mg/día (Tabla 3).

Hubo 44 pacientes (62,9%) que requirieron cambio de modalidad de tratamiento tras alguna monitorización debido a que la Cmin se encontraba fuera de rango. No se observaron diferencias estadísticamente significativas en la probabilidad de requerir cambio de modalidad de tratamiento según la dosis diaria inicial (64,4% para 1.200 mg/día vs 54,5% para 1.800 mg/día; OR=0,66; IC95%: 0,18-2,55).

En dos pacientes se suspendió el linezolid tras la monitorización y en 42 (95,5%) se llevó a cabo el cambio de modalidad. Aunque nueve pacientes (21,4%) requirieron solo cambio de tiempo de infusión, seis (14,3%) solo cambio de frecuencia de administración y uno (2,4%) solo cambio de dosis, el cambio más frecuente afectó a varias de las modalidades mencionadas (n=26; 61,9%). En 18 pacientes se monitorizó la Cmin tras al cambio, logrando que la Cmin se situase en rango en 12 de ellos (66,7%): en cuatro (33,3%) se había realizado un cambio de dosis, frecuencia y tiempo de infusión, en tres (25%) solo un cambio de frecuencia, y en cinco (41,7%) se había realizado un cambio de dos modalidades (dosis, frecuencia, y/o tiempo de infusión).

**Tabla 3:** Resultados en variables PK-PD correspondientes a todas las monitorizaciones realizadas en todos los pacientes durante el tratamiento con linezolid

Variables		Mediana (RIC)
Monitorizaciones por paciente (total)		1 (1)
Aclaramiento de linezolid (mL·kg/min)		2 (2,2)
Volumen de distribución (L/kg)		0,7 (0,4)
Semivida de eliminación (h)		3,5 (2,6)
Constante de eliminación (h^-1^)		0,2 (0,1)
*Objetivos PK/PD*	*Resultado*	*Fuera de rango en algún momento*
	*n (%)*	*n (%)*
Cmin (mg/L)	2,4 (4,1)	26 (37,1)
<2 mg/L		24 (92,3)
>7 mg/L		2 (7,7)
AUC 0-24/CMI (mg·h/L)	62,1 (57,2)	40 (57,1)
<80 mg·h/L		31 (77,5)
>120 mg·h/L		9 (22,5)
T>CMI (%)	86,5 (78,0)	23 (32,9)
<85%		
*Variables en rango terapéutico en la primera monitorización con la pauta inicial de linezolid*		
	*Cmin, AUC 0-24/CMI y T>CMI*	*Cmin*
	*n (%)*	*n (%)*
n (%)	8/43* (18,6)	28/70 (40,0)
*Según dosis total diaria inicial*		
1.200 mg/día	7/8 (87,5)	22/28 (78,6)
1.800 mg/día	1/8 (12,5)	/28 (21,4)
OR (IC95%)	1,11 (0,05-9,08)	2,02 (0,55-7,76)
*Según tiempo de infusión*		
1 h	4/8 (50,0)	/28 (17,9)
3 h	4/8 (50,0)	23/28 (82,1)
OR (IC95%)	0,84 (0,17-4,08)	3,13 (1,05-10,76)**

a: RIC: rango intercuartílico; Cmin: concentración mínima; AUC 0-24: área bajo la curva; CMI: concentración mínima inhibitoria; T>CMI: tiempo por encima de la concentración mínima inhibitoria; OR: odds ratio; IC95%: intervalo de confianza del 95%*: Se disponía del dato de Cmin, AUC 0-24/CMI y T>CMI de linezolid en la primera monitorización con la pauta inicial en 43 pacientes; **: p=0,051 (no estadísticamente significativo por baja potencia)

Veintiún pacientes (30%) recibieron en algún momento del tratamiento una dosis de linezolid superior a la estándar (dos pacientes hasta 2.400 mg/día, y 19 hasta 1.800 mg/día), y tres pacientes recibieron en algún momento dosis inferiores a la estándar. En 53 pacientes (75,7%), el linezolid se infundió durante más de 1 hora en algún momento del tratamiento (en 52 pacientes en 3 horas, y en un paciente en perfusión continua). En dos pacientes se cambió la vía de administración de intravenosa a vía oral.

En la Tabla 4 se muestran los resultados de haber presentado la Cmin en rango terapéutico en la primera monitorización en los principales subgrupos de pacientes (con IMC basal >30 kg/m^2^, con aclaramiento de creatinina >120 mL/min, sometidos a técnicas de reemplazo renal, y menores de 65 años) en función de la dosis diaria inicial de linezolid (1.200 mg frente a 1.800 mg). El porcentaje de pacientes alcanzaron el objetivo de Cmin con la pauta inicial de 1.800 mg/día fue numéricamente superior en comparación con los que recibieron 1.200 mg/día, aunque sin diferencias estadísticamente significativas.

**Tabla 4:** Frecuencia de Cmin de linezolid en rango terapeútico en la primera monitorización según subgrupos de pacientes y en función de la dosis diaria inicial de linezolid

	Cmin de linezolid en rango terapeútico en la primera monitorización				
	Sí		No		p
	n (%)		n (%)		
Linezolid (mg/día)	1.200	1.800	1.200	1.800	
IMC >30 kg/m^2^ (n=31)	9/25	3/6	16/25	3/6	0,65
	(36,0)	(50,0)	(64,0)	(50,0)	
Terapia de reemplazo renal (n=11)	4/7	3/4	3/7	1/4	1
	(57,1)	(75,0)	(42,9)	(25,0)	
Aclaramiento de creatinina >120 mL/min, (n=23)	4/16	3/7	12/16	4/7	0,63
	(25,0)	(42,9)	(75,0)	(57,1)	
<65 años (n=41)	10/31	5/10	21/31	5/10	0,45
	(32,3)	(50,0)	(67,7)	(50,0)	

IMC: índice de masa corporal.

No se observaron diferencias estadísticamente significativas entre los pacientes que presentaron Cmin en rango en algún momento frente a los que no, tanto respecto a los resultados microbiológicos (negativización del cultivo y tiempo hasta la misma, y frecuencia de recaída o nueva infección a 30 días). Respecto a los resultados clínicos, solo se observaron diferencias estadísticamente significativas respecto a la mortalidad a 30 días, que se pierden al ajustar por edad, IMC, y aclaramiento de creatinina (Tabla 5). El análisis de supervivencia reveló que la mortalidad a los 30 días se asoció significativamente con una mayor edad (p=0,003) y un menor aclaramiento de creatinina (p=0,02), lo cual podría ser un reflejo de la propia gravedad basal y del fracaso multiorgánico del paciente crítico.

**Tabla 5:** Resultados microbiológicos y clínicos, global y según Cmin de linezolid en rango terapéutico en algún momento

	Cmin de linezolid en rango terapeútico en algún momento			
	Global	Sí	No	Resultado (IC95%)
*Resultados microbiológicos*				
Negativización del cultivo^a^, n (%)				
	12/31 (38,7)	9/19 (47,4)	3/12 (25,0)	OR=2,70 (0,59 a 15,17)
Tiempo hasta negativización del cultivo (días), media (DE)				
	9 (7,25)	11 (9,0)	6 (4,0)	DM=3,67 (-4,30 a 11,64)
Recaída o nueva infección a 30 días, n (%)				
	24/70 (34,3)	17/41 (41,5)	7/29 (24,1)	OR=2,23 (IC95% 0,80-6,70)
*Resultados clínicos*				
Duración estancia en UCI (días), mediana (RIC):				
	23 (27,8)	24 (34)	17 (17)	DM=0,32 (-14,19 a 14,82)
Duración estancia hospitalaria (días), mediana (RIC):				
	36 (30,5)	37 (41)	33 (21)	DM=-2,78 (-21,85 a 16,29)
Curación clínica^b^, n (%):				
	37/53 (69,8)	22/32 (68,8)	15/21 (71,4)	OR=0,88 (0,25 a 2,90)
Mortalidad a 30 días, n (%):				
	14/70 (20,0)	12/41 (29,3)	2/29 (6,9)	OR=5,59 (1,36 a 38,06); p=0,035
				ORa= 6,90 (1,02 a 142,19)^c^; p=0,093

DM: diferencia de medias; IC95%: intervalo de confianza del 95%; OR: *odds ratio*; RIC: rango intercuartílico; a: solo se consideran los pacientes que inicialmente tenían al menos un aislamiento de un microorganismo sensible a linezolid (n=31); b: ausencia de fiebre y normalización de parámetros inflamatorios en los pacientes que al inicio del tratamiento presentaban fiebre y/o alteración de parámetros inflamatorios; c: OR ajustada por edad, índice de masa corporal y aclaramiento de creatinina.

La Tabla 6 muestra la relación entre las características demográficas y clínicas basales de los pacientes y distintas variables de resultado. La mayoría de relaciones fue no significativa, a excepción de un mayor cumplimiento de Cmin en pacientes no inmunodeprimidos, y un menor aclaramiento de creatinina y mayor edad en pacientes que fallecieron en los 30 días posteriores.

**Tabla 6:** Relación de las características demográficas y clínicas basales de los pacientes con el cumplimiento de objetivos en parámetros PK-PD en la primera monitorización de linezolid con la pauta inicial, y con los resultados microbiológicos y clínicos

	Cumplimiento de objetivos en parámetros PK-PD	
	Cmin, AUC 0-24/CMI y T>CMI	Cmin
	Sí (n=8)	Sí (n=28)
	No (n=35)	No (n=42)
	p	p
Edad (años), mediana (RIC)	62,0 (55,5-66,8)	64,0 (54,0-71,0)
	59,0 (51,5-69,5)	59,0 (50,2-69,8)
	0,79	0,38
Sexo (mujer), n (%)	4 (50,0)	9 (32,1)
	15 (42,9)	16 (38,1)
	1	0,80
IMC, mediana (RIC)	33,1 (30,2-36,0)	31,0 (27,8-36,9)
	29,9 (26,3-34,7)	31,6 (26,4-34,7)
	0,34	0,52
Terapia de reemplazo renal, n (%)	4 (50,0)	7 (25)
	7 (20,0)	4 (9,5)
	0,17	0,06
Aclaramiento creatinina (mL/min)*, mediana (RIC)	93,3 (79,3-109,0)	81,8 (71,6-123,0)
	105,0 (63,9-140,0)	102,0 (66,0-139,0)
	0,76	0,50
BRt >1,2 mg/dL y/o TA >3LSN, n (%)	4 (50,0)	14 (50,0)
	7 (20,0)	11 (26,2)
	0,17	0,07
Inhibidores potentes del CYP3A4, n (%)	5 (62,5)	9 (32,1)
	11 (31,4)	9 (21,4)
	0,12	0,40
Inductores potentes del CYP3A4, n (%)	0	1 (3,6)
	1 (2,9)	3 (7,1)
	1	0,64
Inmunodepresión, n (%)	1 (12,5)	1 (3,6)
	8 (22,9)	9 (21,4)
	1	0,04
Shock séptico, n (%)	3 (37,5)	14 (50,0)
	14 (40,0)	18 (42,9)
	1	0,63
*Variables*	*Resultados microbiológicos*	*Resultados clínicos*	
	*Recaída/nueva infección a 30 días*	*Curación clínica*	*Mortalidad a 30 días*
	*Sí (n=24)*	*Sí (n=37)*	*Sí (n=14)*
	*No (n=46)*	*No (n=16)*	*No (n=56)*
	*p*	*p*	*p*
Edad (años), mediana (RIC)	62,5 (55,5-69,5)	58,5 (52,5-70,0)	71,5 (67,2-75,0)
	59,5 (51,0-70,0)	68,0 (56,0-74,0)	58,0 (50,8-68,2)
	0,62	0,13	0,003
Sexo (mujer), n (%)	10 (41,7)	13 (35,1)	4 (28,6)
	15 (32,6)	5 (31,3)	21 (37,5)
	0,63	0,80	0,75
IMC, mediana (RIC)	30,5 (27,8-38,2)	32,3 (28,0-34,7)	27,5 (20,5-33,2)
	31,8 (26,3-34,7)	27,5 (23,3-34,7)	31,9 (27,9-35,1)
	0,26	0,15	0,07
Terapia de reemplazo renal, n (%)	6 (25)	8 (21,6)	2 (14,3)
	5 (10,9)	1 (6,25)	9 (16,1)
	0,08	0,25	1
Aclaramiento creatinina (mL/min)*, mediana (RIC)	92,1 (72,5-127,0)	81,7 (63,9-129,0)	68,2 (50,1-89,6)
	97,1 (66,0-136,0)	82,6 (52,4-124,0)	117,0 (73,0-138,0)
	0,78	0,66	0,02
BRt >1,2 mg/dL y/o TA >3LSN, n (%)	7 (29,2)	15 (40,5)	7 (50)
	18 (39,1)	4 (25)	18 (32,1)
	0,57	0,77	0,35
Inhibidores potentes del CYP3A4, n (%)	7 (29,2)	10 (27,0)	5 (35,7)
	11 (23,9)	4 (25)	13 (23,2)
	0,85	1	0,54
Inductores potentes del CYP3A4, n (%)	0	1 (2,7)	1 (7,1)
	4 (8,7)	1 (6,25)	3 (5,4)
	0,29	0,24	1
Inmunodepresión, n (%)	2 (8,3)	3 (8,1)	3 (21,4)
	8 (17,4)	2 (12,5)	7 (12,5)
	0,48	0,37	0,41
Shock séptico, n (%)	7 (29,2)	22 (59,5)	7 (50)
	25 (54,3)	7 (43,8)	25 (44,6)
	0,08	0,63	0,95

Cmin, AUC 0-24/CMI y T>CMI; RIC: rango intercuartílico; IMC: índice de masa corporal (kg/m^2^); *: aclaramiento de creatinina (mL/min) en pacientes sin terapia de reemplazo renal (n=59); BRt: bilirrubina total; TA: transaminasas; LSN: límite superior de la normalidad.

## DISCUSIÓN

Más del 80% de pacientes críticos en este estudio no alcanzaron los objetivos PK/PD integrales en la primera monitorización con la pauta inicial de linezolid. Aunque el 40% presentó una Cmin en rango terapéutico con la pauta inicial, estos resultados refuerzan la evidencia previa que califica las dosis estándar de linezolid como insuficientes en el entorno crítico. Y ello a pesar de que casi un tercio de los pacientes recibieron una dosis de linezolid superior a la estándar en algún momento del tratamiento y que la duración de la infusión se extendió en tres cuartas partes de pacientes, lo cual refleja la frecuencia con que las pautas convencionales resultan insuficientes para alcanzar objetivos PK/PD. Esta infraexposición sistemática coincide con lo reportado en estudios previos donde el mayor aclaramiento plasmático en pacientes críticos compromete la eficacia de la dosis de 600 mg/12h^12^^-^^15^. Además, nuestro análisis muestra que la monitorización aislada de la Cmin podría sobreestimar la adecuación del tratamiento si no se consideran el AUC/CMI y el T>CMI (18,6%).

Una revisión sistemática publicada en 2022 concluyó que la administración intermitente de linezolid puede resultar subóptima para alcanzar los objetivos PK-PD en pacientes críticos^15^. Por ello, se sugiere administrar linezolid en infusión intravenosa continua como estrategia para mantener las concentraciones plasmáticas por encima de la CMI durante más tiempo^16^^,^^17^. Varios estudios han demostrado que la infusión continua se asocia con tasas de curación clínica más rápidas y superiores, y a menor riesgo de trombocitopenia^11^ y de mortalidad en comparación con la infusión intermitente^18^^,^^19^. Aun así, la dosificación de linezolid en infusión continua debe estar guiada por TDM^20^^,^^21^.

La alta tasa de pacientes que requirieron ajustes en la modalidad de tratamiento pone de manifiesto la extrema variabilidad farmacocinética intra e interindividual de la población crítica. Nuestros hallazgos, en los que dos tercios de los pacientes alcanzaron el rango terapéutico tras el ajuste guiado, respaldan la monitorización terapéutica (TDM) como una herramienta indispensable para corregir la exposición subóptima. Aunque se llevaron a cabo modificaciones en la mayoría de pacientes (96%), la Cmin se revaluó tras el ajuste en menos de la mitad de los casos (43%) pacientes, con éxito en dos tercios de ellos. Estos hallazgos sugieren que la monitorización secuencial puede ser una herramienta efectiva para optimizar la exposición al fármaco, pero también que en la práctica clínica no siempre se completa de forma sistemática.

Aunque no se observaron diferencias estadísticamente significativas, el estudio ha permitido detectar posibles grupos de riesgo que podrían beneficiarse del empleo de dosis iniciales superiores a la estándar para alcanzar los objetivos PK/PD más rápidamente, como pacientes con IMC >30 kg/m^2^, con aclaramiento de creatinina >120 mL/min, sometidos a técnicas de reemplazo renal y menores de 65 años. Nuestro estudio aporta valor al identificar estos subgrupos en un contexto de práctica clínica real, sugiriendo que la optimización del tratamiento debe ir más allá de la dosis, integrando también la prolongación de la infusión para maximizar el tiempo por encima de la CMI^9^^,^^10^^,^^21^.

El diseño observacional y retrospectivo, sumado a un tamaño muestral limitado, ha condicionado la potencia estadística necesaria para identificar diferencias significativas en la consecución de los objetivos PK/PD entre las distintas modalidades de tratamiento. No obstante, hemos podido documentar la dificultad para alcanzar los objetivos con la pauta estándar. Se recogió la información registrada en la historia clínica en el contexto de la práctica clínica real, lo que conlleva falta de información en ciertas variables. En cuanto a la toxicidad de linezolid, principalmente hematológica (trombocitopenia y anemia), no fue posible analizarla, debido al carácter retrospectivo del estudio y a que dicha toxicidad es de carácter multifactorial en el paciente crítico, lo que impide atribuir causalidad.

La ausencia de una relación clara entre los niveles de fármaco y los resultados clínicos finales concuerda con la naturaleza multifactorial del paciente crítico, donde la gravedad basal (reflejada en puntuaciones SOFA elevadas) y el control del foco infeccioso pueden enmascarar el impacto directo de la optimización antibiótica.

Entre las fortalezas del estudio, destaca que se trata de un estudio de práctica clínica real en pacientes ingresados en una UCI de un hospital universitario, que permite la extrapolación de los hallazgos a otras unidades análogas de otros hospitales. Por otro lado, el estudio analiza el potencial impacto de las distintas modalidades de tratamiento y de la TDM en los parámetros PK-PD, así como en variables microbiológicas y clínicas relevantes, dando una visión global del potencial efecto a distintos niveles.

En conclusión, menos del 20% de los pacientes alcanzó todos los objetivos farmacocinéticos/ farmacodinámicos con la pauta inicial. Estos resultados sugieren la necesidad de individualizar y monitorizar las pautas para optimizar el tratamiento en esta población, especialmente en perfiles de riesgo.

Consideramos que a pesar de su baja potencia estadística, este estudio puede servir como base para investigaciones futuras con mayor tamaño muestral y diseños más robustos, idealmente ensayos clínicos aleatorizados, que permitan establecer conclusiones firmes sobre el impacto de las distintas modalidades de tratamiento y de la TDM de linezolid en resultados microbiológicos y clínicos en pacientes críticos, así como sobre los perfiles de pacientes que podrían beneficiarse de pautas intensificadas.

## Data Availability

Se encuentran disponibles bajo petición a la autora de correspondencia.
